# Questionnaire Survey on the Current Situation and Experience in Prevention and Control Measures at Urology Clinics During the COVID-19 Epidemic in China

**DOI:** 10.3389/fpubh.2021.670889

**Published:** 2021-08-20

**Authors:** Xiao-Liang Zhu, Hai-Hong Jiang, Ming-Hui Jiang, Wen-Li Liu, Zi-Lu Sheng, Jia-Hui Liu, Meihao Wang

**Affiliations:** ^1^Department of Urology, Taizhou Hospital of Zhejiang Province Affiliated to Wenzhou Medical University, Wenzhou, China; ^2^Department of Urology, The First Affiliated Hospital of Wenzhou Medical University, Wenzhou, China; ^3^Department of Radiology, The First Affiliated Hospital of Wenzhou Medical University, Wenzhou, China

**Keywords:** COVID-19, experience, SARS-CoV-2, questionnaire, urological outpatient, epidemic

## Abstract

COVID-19, the coronavirus disease 2019; SARS-CoV-2, the coronavirus 2; ACE2, angiotensin converting enzyme 2; S protein, spiked glycoprotein; TMPRSS2, transmembrane serine protease 2; WHO, World Health Organization.

**Purpose:** Although the coronavirus disease 2019 (COVID-19) pandemic, caused by the severe acute respiratory syndrome coronavirus 2, has been viably controlled in China, a new normal in healthcare strategies has become standard in China and worldwide. We conducted a questionnaire study to disseminate the experience from China in terms of urology outpatient prevention and control measures under standardized prevention policies against COVID-19.

**Participants and Methods:** From May 3, 2020 to June 25, 2020, we conducted an anonymous cross-sectional questionnaire study, focused on the status of and experiences with outpatient urology prevention and control measures during the COVID-19 pandemic. The targeted respondents were urologists in mainland China, covering all levels of hospitals and clinics.

**Results:** A total of 216 (97%) valid responses were collected. We found that 183 (85%) respondents were from outside of Hubei province in China. One-hundred-and-fifty-eight (73%) respondents believed that SARS-CoV-2 could be detected in urine, and that protection against urine exposure was needed. Over 80% of respondents recommended WeChat application or similar online video meetings for virtual outpatient consultations. The suggested flowcharts and recommendations to prevent new cases were easy to understand and approved by most physicians, which could provide reference for outpatient prevention and control. We still need to make adequate preparations under the new normal of the COVID-19 Epidemic, especially for those suspected of being infected.

**Conclusions:** Although the scientific validation of the questionnaire is limited, it provides a first snapshot of the experiences relating to the prevention and control measures in urology clinics in China, and can inform future policies in this field.

## Introduction

The coronavirus disease 2019 (COVID-19) began at the end of 2019 and has rapidly become an ongoing global pandemic. The COVID-19 pandemic has major ramifications for global health and the economy (1, 2). To control the spread of the epidemic, the Chinese Government and people have put forth unremitting efforts, which proved to be effective so far (3). COVID-19 prevention and control measures have become standard in Chinese healthcare providers practice due to the difficult situation of both an ongoing global pandemic and sporadic domestic epidemics. The recommended preventive measures include social distancing, wearing face masks, hand washing, disinfecting surfaces, and isolation for people exposed (4). The COVID-19 caused by severe acute respiratory syndrome coronavirus 2 (SARS-CoV-2) has highly variable symptoms ranging from almost none to life-threateningly severe (5, 6). It has been confirmed that SARS-CoV-2 can be transmitted by droplets and close contact. Additionally, SARS-CoV-2 is detectable in urine, as it becomes concentrated in the urinary system and in fecal matter, remaining positive in the excretion of some patients who have recovered from COVID-19 (7). A systematic review of COVID-19 and its potential urological manifestations revealed that 5.74% urine samples from COVID-19 patients were positive for viral RNA, but the duration of viral shedding in urine was unknown (8). A global survey on the impact of COVID-19 on urological services showed that 41% of respondents reported that their hospital staff had been diagnosed with COVID-19 infection (2). To this end, we conducted a national questionnaire survey to disseminate the Chinese urology outpatient experience with prevention and control measures under the new normality of COVID-19. We hope our results and experiences can help guide our future outpatient work, and can also help international counterparts with anti-COVID-19 efforts in urological outpatient practice.

## Participants and Methods

### Study Design and Population

To best represent the current situation in prevention and control measures in urology outpatient practice in China, during the COVID-19 pandemic, the targeted respondents were urologists in mainland China, covering five levels of hospitals and clinics from 22 provinces, two autonomous regions and four municipalities (Table 1). We conducted an anonymous cross-sectional questionnaire study targeting domestic urologists in China. Questionnaires were distributed by email and the WeChat application from May 3, 2020 to June 25, 2020. WeChat is one of most popular social applications, with over one billion users and over 400 million active users daily.

**Table 1:** Demographics of survey respondents.

**Variables**	***n***	**%**
	(N=216)	
**1. Title (years in practice)**
Chief physician	69	32.0
Associate chief physician	78	36.0
Attending physician	42	19.5
Resident physician	19	8.8
Others	8	3.7
**2. Types of hospital/institution**
Level III-A hospital	138	64.0
Level III-B hospital	27	12.5
Level II hospital	43	19.9
Level I hospital	5	2.3
Others	3	1.3
**3. Region**
ZheJiang province	47	21.8
HuBei province	33	15.3
Beijing municipal	13	6.0
GuangDong province	12	5.6
HeBei province	10	4.6
ShanDong province	9	4.2
ShanXi province	8	3.7
HeNan province	8	3.7
HuNan province	8	3.7
AnHui province	7	3.2
HeiLongJiang province	6	2.8
SiChuan province	6	2.8
LiaoNing province	5	2.3
JiLin province	5	2.3
JiangSu province	5	2.3
FuJian province	5	2.3
ChongQing	5	2.3
JiangXi province	3	1.4
HaiNan province	3	1.4
GuiZhou province	3	1.4
Inner Mongolia autonomous Region	3	1.4
YunNan province	2	0.9
ShanXi province	2	0.9
GanSu province	2	0.9
TianJin	2	0.9
ShangHai	2	0.9
QingHai province	1	0.5
Ningxia Hui Autonomous Region	1	0.5

### The Design and Contents of the Questionnaire

The questionnaire comprised a total of 34 questions, with single-choice and multiple-choice response items. The content of the questionnaire was mainly based on the seventh edition of the “Chinese Novel Coronavirus Diagnosis and Treatment Guide for Pneumonia,” which was relatively authoritative in China at the beginning of the COVID-19 outbreak (9). The questionnaire was also referred to published scientific articles of the latest research on COVID-19 at the beginning of the COVID-19 outbreak (10–12). Considering that our understanding of this disease was limited, most of the questions designed were in non-scale form. Although the questionnaires have had passed the reliability and validity test according to The Statistical Program for Social Sciences software analysis, the scientific evidence upon which the survey was based was still limited. The entire questionnaire is presented in Supplementary Tables 1–6.

### Data Analysis

We randomly distributed 250 questionnaires *via* email and the WeChat application to urologists who had “urology” listed as their specialty. A total of 222 (89%) questionnaires were returned. The number of 250 questionnaires was expected to provide adequate data with appropriate statistical power and workload with optimized representative domestic professional scale. After excluding questionnaires from non-urologist and incomplete responses, 216 (97%) responses were confirmed as valid. For each question in the questionnaire, the number of participants and the corresponding percentage was calculated for each of the response items.

## Results

### Demographics

The results of this questionnaire represented the views of physicians mainly outside Hubei Province in China (Table 1). The respondents comprised of urological doctors working at various hospital levels, from senior urologists to junior urological specialists. Physicians from various provinces of China completed the questionnaire, including physicians from the Hubei province and its capital city, Wuhan, where the first reported and confirmed COVID-19 cluster cases were location (9).

### SARS-CoV-2 and the Genitourinary System

Most respondents indicated that they believed that SARS-CoV-2 could be detected in urine, while only some believed that SARS-CoV-2 was detectable in semen (Table 2.1.1,2.1.2). Regarding transmission methods, only 35 (16%) respondents believed that SARS-CoV-2 could be spread through the urogenital system (Table 2.1.3). Over 152 (70%) respondents believed that the kidney, bladder, and testes could be affected by SARS-CoV-2 (Table 2.1.4).

**Table 2:** The main results of the questionnaire survey.

**1. SARS-CoV-2 and the genitourinary system**	***n* (%)**
***1.1 Did you think the SARS-CoV-2 can be detected in the urine?***	
**Yes**	**158 (73%)**
**No**	**34 (16%)**
**Not sure**	**24 (11%)**
***1.2 Did you think the SARS-CoV-2 can be detected in the semen?***	
**Yes**	**19 (9%)**
**No**	**56 (26%)**
**Not sure**	**141 (65%)**
***1.3 Did you know how the SARS-CoV-2 may be transmitted? (multiple-choice response items)***	
**Droplet transmission**	**214 (99%)**
**Close contact transmission**	**210 (97%)**
**Aerosol transmission**	**184 (85%)**
**Blood transmission**	**39 (18%)**
**Semen transmission**	**35 (16%)**
***1.4 What organs in the genitourinary system did you think may be affected by SARS-CoV-2? (multiple-choice response items)***	
**Kidney**	**163 (75%)**
**Testis**	**182 (84%)**
**Epididymis**	**123 (57%)**
**Bladder**	**167 (77%)**
**Seminal vesicle**	**117 (54%)**
**Prostate gland**	**87 (40%)**
**2. Impact of COVID-19 on outpatient clinics**	
***2.1 Which were the recommended virtual outpatient consultation ways during the epidemic? (multiple-choice response items)***	
**Telephone**	**118 (55%)**
**WeChat or video**	**175 (81%)**
**Third Party APP**	**72 (33%)**
***2.2 During the epidemic, did you think the online virtual outpatient service was better than the routine outpatient service?***	
**Yes**	**118 (55%)**
**No**	**66 (30%)**
**Not sure**	**32 (15%)**
**3. Impact of COVID-19 on psychological status**	
***3.1 During the epidemic, how did you feel when you came in close contact with outpatient patients, especially those suspected of COVID-19? (multiple-choice response items)***	
**Tired**	**130 (60%)**
**Anxious**	**91 (42%)**
**Delayed or resisted**	**75 (35%)**
**Confused**	**29 (13%)**
**Easily excited**	**53 (25%)**
**Depressed**	**45 (21%)**
**Loss of attention and memory**	**49 (23%)**
**Poor sleep and even insomnia**	**49 (23%)**
**Poor or increased appetite**	**14 (6%)**
**Nothing changed**	**10 (5%)**
***3.2 During the epidemic, did any patients experience any of the following negative emotions as a result of the epidemic? (multiple-choice response items)***	
**Tired**	**109 (50%)**
**Anxious**	**118 (55%)**
**Delayed or resisted**	**46 (21%)**
**Confused**	**115 (53%)**
**Easily excited**	**47 (22%)**
**Depressed**	**68 (31%)**
**Loss of attention and memory**	**24 (11%)**
**Poor sleep and even insomnia**	**96 (44%)**
**Poor or increased appetite**	**18 (8%)**
**Nothing changed**	**15 (7%)**
***3.3 Did you observe any urinary symptoms or exacerbation of urinary symptoms due to mood changes caused by the epidemic during the outpatient? (multiple-choice response items)***	
**Frequent micturition**	**110 (51%)**
**Nocturia**	**65 (29%)**
**Pain in the penis or burning sensation in the urethra**	**24 (11%)**
**Erectile dysfunction**	**31 (14%)**
**Premature ejaculation**	**17 (8%)**
**Pain and discomfort in the perineum**	**42 (19%)**
**Discomfort and pain in the testicles or perineum**	**29 (13%)**
**Nothing changed**	**71 (33%)**
***3.4 During the epidemic, was there a need for psychosocial support for outpatient health workers or patients?***	
**Yes**	**178 (82%)**
**No**	**10 (5%)**
**Not sure**	**28 (13%)**
**4. Experience with COVID-19 screening in outpatient clinics**	
***4.1 During the epidemic, what did you consider to be the main bases of importance for the initial determination of patients with suspected COVID-19 in outpatient clinic? (multiple-choice response items)***	
**Consultation of epidemiological history**	**176 (81%)**
**Measurement of body temperature**	**110 (51%)**
**Coughs and other respiratory symptoms**	**76 (35%)**
**Examination of chest CT**	**152 (70%)**
**Blood routine, CRP, blood pressure test and so on**	**68 (31%)**
**Antibody or nucleic acid test for SARS-CoV-2 virus**	**84 (40%)**
***4.2 What preventive and control measures did your urology outpatient take during the epidemic? (multiple-choice response items)***	
**wore a surgical mask**	**199 (93%)**
**Wore gloves and disinfected hands**	**149 (69%)**
**Disinfected the clinic environment**	**129 (60%)**
**Wore protective goggles**	**119 (55%)**
**Wore a protective surface screen**	**96 (44%)**
**Wore simple protective clothing**	**84 (39%)**
**Wore standard full-body protective clothing**	**32 (15%)**
**Patients were accompanied by family members**	**84 (39%)**
**Patients were unaccompanied by family members and alone**	**94 (42%)**
***4.3 In light of the current prevention policy, which groups of individuals should be cautious in urological outpatient? (multiple-choice response items)***	
**Urinary catheterization**	**135 (63%)**
**Prostate palpation and treatment**	**85 (39%)**
**Collection of patient secretions**	**96 (44%)**
**Bladder perfusion**	**111 (51%)**
**Urodynamics-related tests**	**51 (24%)**
**Replacement of stoma fistula**	**97 (45%)**
**Replacement of stoma pockets**	**42 (19%)**
***4.4 What were the main important outpatient procedures that required increased protection during the epidemic? (multiple-choice response items)***	
**Circumcision**	**86 (40%)**
**Cystoscopy**	**169 (78%)**
**Urethral stent removal or replacement**	**145 (67%)**
**Extracorporeal vibration lithotripsy**	**133 (62%)**
**Urethral dilation**	**93 (43%)**
**Resection of local superficial lump**	**40 (19%)**
**5. Experience of patients with a history of COVID-19 in outpatient clinics**	
***5.1 Did patients with history of COVID-19 need to be examined separately? And did the medical waste need to be disposed separately?***	
**Yes**	**178 (82%)**
**No**	**28 (13%)**
**Not sure**	**10 (5%)**
***5.2 What else should be done in the urology outpatient clinic for the patients who recovered from COVID-19? (multiple-choice response items)***	
**Detection of SARS-CoV-2 in urine and semen**	**119 (55%)**
**Testicular and semen screening for fertility in men of reproductive age**	**126 (58%)**
**Ultrasound of the urinary system**	**58 (27%)**
**CT examination of the urinary system**	**44 (20%)**
**There was no need to specifically check the urinary system**	**50 (23%)**

### Impact of COVID-19 on Outpatient Clinics

One-hundred-and-seventy-five (81%) respondents recommended WeChat or similar internet video meetings as virtual outpatient consultations during the epidemic (Table 2.2.1). One-hundred-and-eighteen (55%) respondents believed that these virtual visits were better than routine service for the prevention of new COVID-19 cases (Table 2.2.2).

### Impact of COVID-19 on Psychological Status

According to the results of the questionnaire, both the respondents and their patients have been significantly affected by the COVID-19 epidemic, mainly in terms of weariness and anxiety (Table 2.3.1, 2.3.2). One-hundred-and-seventy-eight (82%) respondents believed that appropriate psychological consultations are essential for supporting their outpatient practice (Table 2.3.4). In addition, 110 (51%) respondents indicated that their patients experienced significant frequent urination symptoms during the epidemic (Table 2.3.3). In addition to medical issues, changes in mental health status may affect patients' lower urinary tract symptoms, especially in terms of frequency.

### Experience With COVID-19 Screening in Outpatient Clinics

According to the results of the questionnaire, epidemiological history and chest computed tomography (CT) examination were considered the most important screening methods (Table 2.4.1). The majority of respondents used the following preventive measures in their outpatient practice: wearing a surgical mask [199(93%)], wearing gloves and performing hand sterilization [149(69%)], and environmental disinfection [149(69%)] (Table 2.4.2). The majority of respondents believed that catheterization [135(63%)] and intravesical installation [111(51%)] were the main procedures requiring precautions to be taken during outpatient treatments (Table 2.4.3). During outpatient surgery, cystoscopy, ureteral stent removal or replacement, and extracorporeal shock wave lithotripsy were the primary concerns (Table 2.4.4). In these procedures, the operator is likely to be in close contact with patients and the patients' urine.

**Figure 1:**
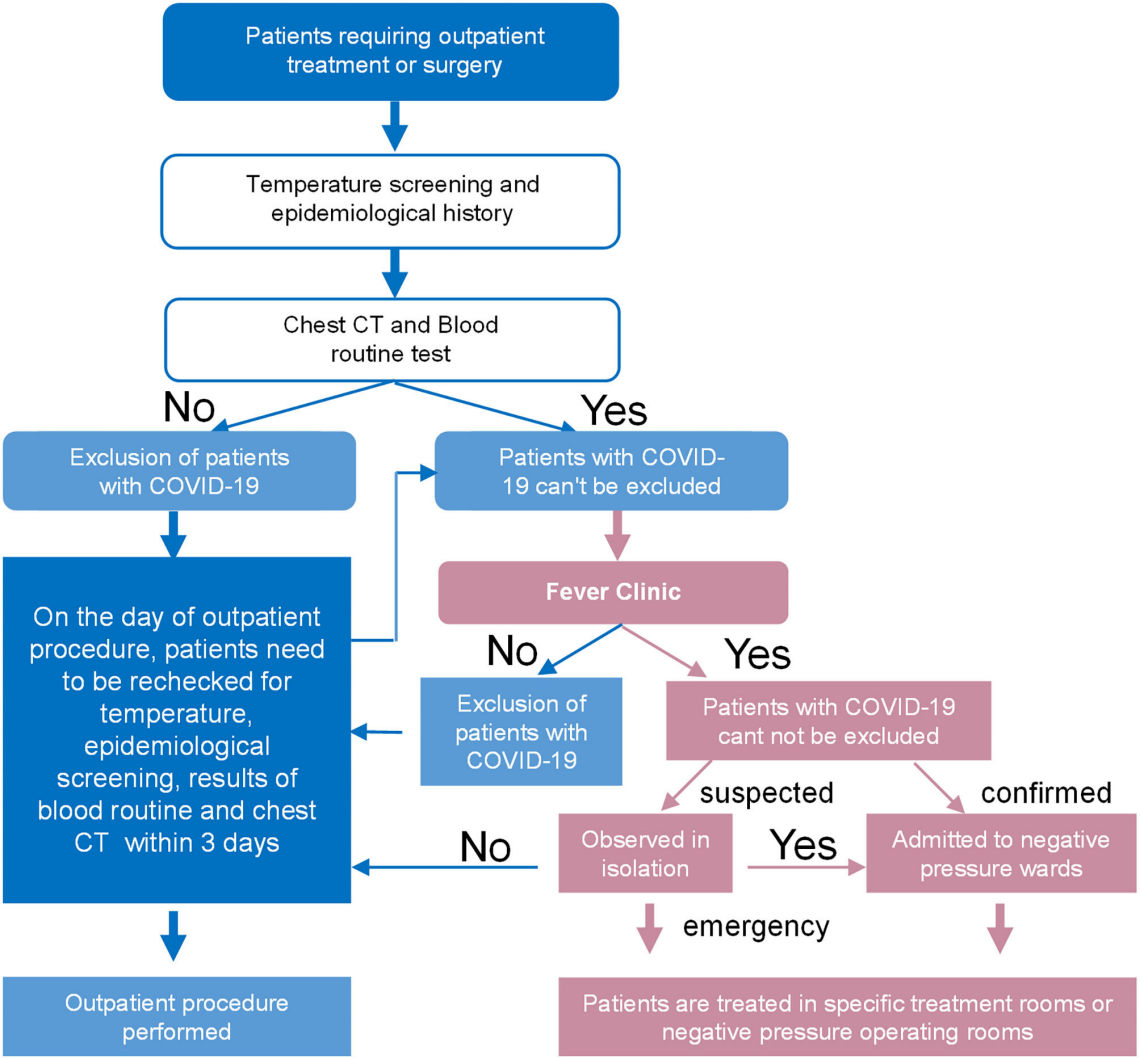
Outpatient procedures flowchart for screening patients with COVID-19.

**Figure 2:**
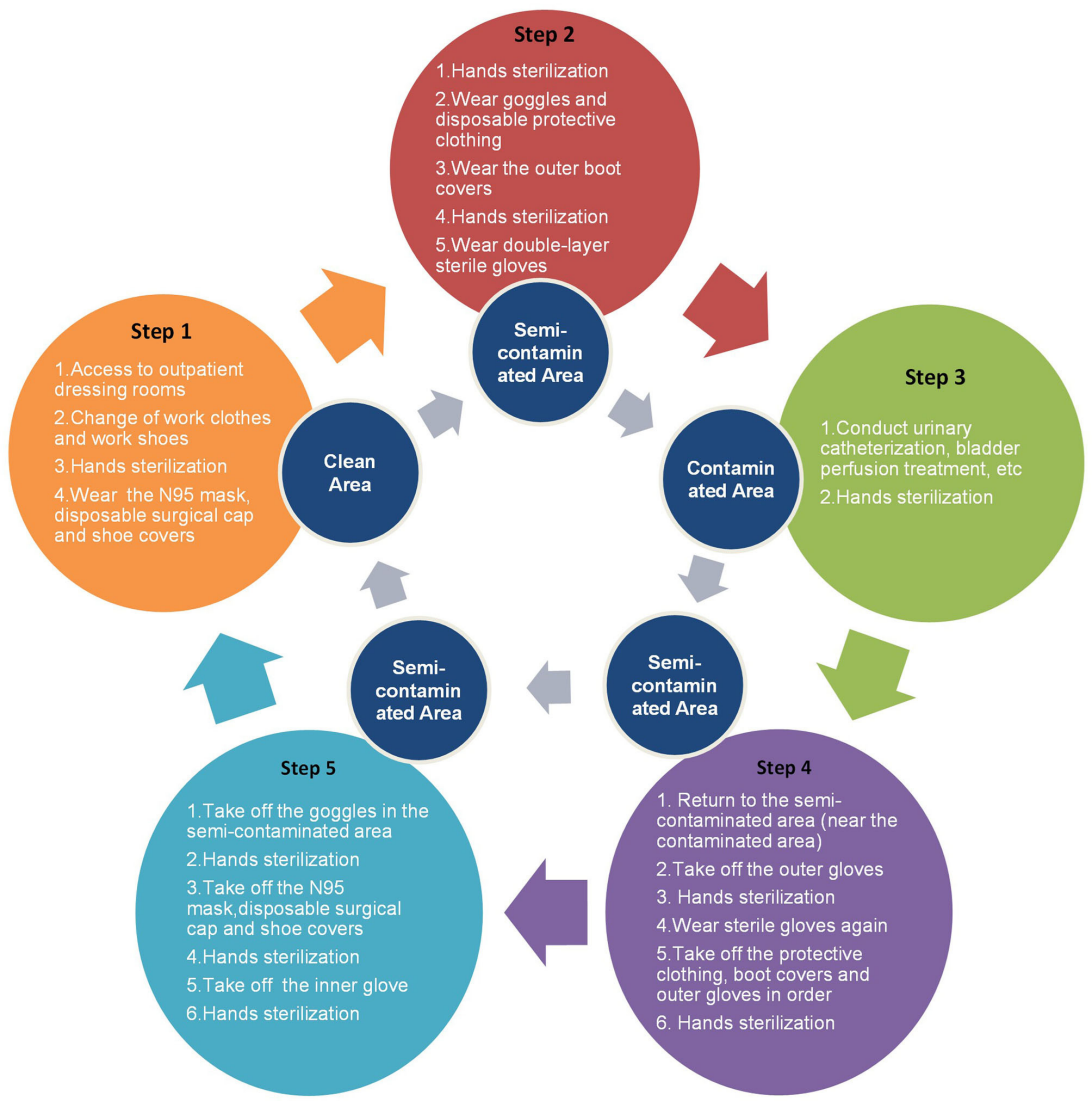
Flowchart for wearing protection in outpatient treatment rooms.

**Figure 3:**
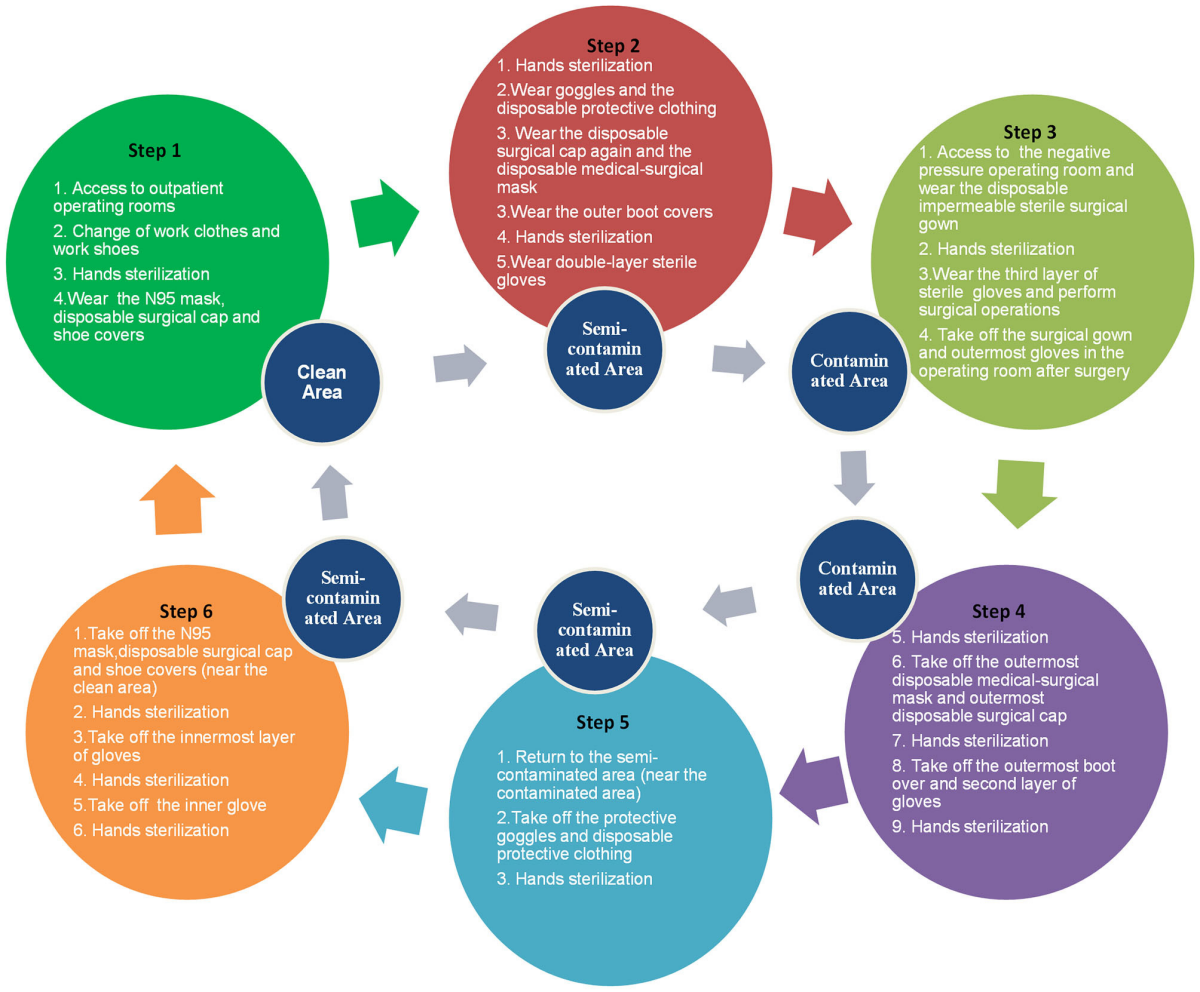
Flowchart for wearing protection in outpatient operating rooms.

The flowcharts we provided were overwhelmingly recognized and approved by the respondents, and included an outpatient procedures flowchart for screening patients with COVID-19 (Figure 1), a flowchart for wearing protection in outpatient treatment rooms (Figure 2), a protection flowchart for wearing protection in outpatient operating rooms (Figure 3), and a table of recommendations for protective options in urological outpatient clinics (Table 3).

**Table 3:** Recommendations for protective options in urological outpatient clinics.

**—**	**Hands sterilization**	**Disposable medical-surgical mask**	**N95 mask**	**Protective goggles**	**Latex gloves**	**Disposable isolation garment**	**Protective clothing**	**Disposable medical cap**	**Disposable medical shoe covers**	**Work clothing**
Protection of outpatient staff	√	√	S	S	√	N	N	√	N	√
Outpatient surgical procedure protection against non-confirmed or suspected COVID-19 patients	√	√	S	S	√	S	N	√	N	√
Outpatient surgical protection against confirmed or suspected COVID-19 patients	√	N	√	√	√	N	√	√	√	√
Protection against the transfer of confirmed or suspected patients' contamination	√	N	√	S	√	√	S	√	√	√

*√ indicates personal protective equipment is recommended. S indicates personal protective equipment is a selective option; N indicates no recommendation*.

### Experience With Patients With a History of COVID-19 in Outpatient Clinics

When treating patients who have recovered from COVID-19, the majority of respondents stated that it is still necessary to examine such patients separately, and that their medical waste should be separated in outpatient clinics (Table 2.5.1). Most respondents recommended testicular and semen screening for fertility in men of reproductive age who have recovered from COVID-19 (Table 2.5.2).

## Discussion

### SARS-CoV-2 and the Genitourinary System

COVID-19 has been found to infect human respiratory epithelial cells *via* a molecular mechanism of spiked glycoprotein (S protein) interaction with human angiotensin converting enzyme 2 (ACE2) receptor, causing lung tissue damage (13). Transmembrane serine protease 2 (TMPRSS2) appears to be the progenitor of the S protein that enhances ACE2 receptor-mediated viral entry (14). Utilizing the latest single-cell RNA sequencing data, a research team from Shanghai Jiaotong University analyzed ACE2 receptor expression in relevant organs and cell types of major human physiological systems and found that the heart, esophagus, kidney, bladder, and ileum all have ACE2 receptor expression similar to or higher than that in alveoli (15). Additionally, a research team from Suzhou Hospital affiliated with Nanjing Medical University analyzed the expression of ACE2 receptor in different human organs using existing datasets and found that ACE2 receptor is highly expressed in renal tubular cells, testicular mesangial cells, and seminiferous tubular cells of the testis (16). The above findings imply that, at the RNA level, the kidneys, bladder, and testes of the urinary system are potential target organs for SARS-CoV-2, concordant with the beliefs of the respondents in the current study.

Furthermore, Guan et al. (10) confirmed that SARS-CoV-2 can be detected in excreta such as urine and feces. Consequently, whether sexual contact is one of the potential routes of SARS-CoV-2 transmission has been raised (17). Li et al. (18) found that SARS-CoV-2 was detected in the semen of 6 out of 38 patients diagnosed with COVID-19. Interestingly, Pan et al. (19) did not detect SARS-CoV-2 in the semen of 34 patients diagnosed with COVID-19 in Wuhan, China. Therefore, we suspect that the detectability in semen might be related to viral load, virus survival time, and the blood-testis barrier. Currently, there is no direct evidence of this mode of transmission. Accordingly, our questionnaire study found that less than half of respondents believed that SARS-CoV-2 is detectable in semen. However, Corman et al. (20) suggested that RNAemia is not equivalent to infectiousness. Additionally, there is a lack of direct evidence that SARS-CoV-2 can be transmitted by blood transfusion (21).

Acute kidney injury has been reported to be a common complication among hospitalized patients with COVID-19 (22). It is considered a marker of disease severity and a negative prognostic factor for survival in patients with COVID-19, based on experience in the United States (23). It was still unclear whether SARS-CoV-2 directly affect the kidney, although renal histopathological analyses of 26 Chinese COVID-19 patients suggested varying degrees of acute tubular injury (24). The prognosis of COVID-19 patients with acute kidney injury was poor, especially those patients with other underlying diseases (25, 26).

### Impact of COVID-19 on Outpatient Clinics

According to the local public health department advice, most people chose to stay at home during the epidemic, especially at the beginning of the outbreak. In order to effectively maintain close contact and communication with patients, physicians and patients stayed in touch *via* WeChat, video calls, and telephone. A global internet survey revealed that the use of telemedicine related devices by urologists nearly tripled during the COVID-19 epidemic (27). This was indeed a pragmatic approach to reducing the risk of transmission, and was worth promoting. Most patients have already benefited from virtual outpatient consultations. However, treatment during the epidemic was compromised for patients with primary symptoms of urinary tumors and post-operative patients with urinary tumors, which would affect their survival over time.

### Impact of COVID-19 on Psychological Status

Psychological factors, such as psychological stress and depression, clearly affect the human endocrine and immune systems (28). Psychological factors can cause metabolic dysregulation of neurotransmitters, such as monoamines and peptides, resulting in hypothalamic-pituitary-gonadal axis and hypothalamic-pituitary-adrenal axis dysfunction, which in turn affects the endocrine and immune reproductive system functions in human, resulting in reproductive dysfunction (29). The psychological states of stress and anxiety experienced by patients with chronic prostatitis are considered an important factor in the development or exacerbation of inflammation. A global survey of psychological impact among surgical providers showed that the COVID-19 pandemic may have a mental health legacy outlasting its course (30). Based on the results of our questionnaire study, the psychological status of both clinical staff and patients have been impacted by the epidemic to varying degrees, and both clinical staff and patients need opportune mental help. Above all else, patients should be effectively managed to obtain accurate healthcare information from the World Health Organization (WHO) internationally and the domestic National Health Care Commission (31, 32), so as to not be impacted by rumors and to not spread inaccurate data. These recommendations can still benefit those patients who need assistance during the COVID-19 pandemic.

### Experience With COVID-19 Screening in Outpatient Clinics

With the continuous importation of COVID-19 cases from outside the country (33–35), and the increasingly subtle epidemiological history and atypical clinical manifestations (36), additional cases will appear in outpatient clinics for two or three generations (37). The performance of PCR-based diagnostic depended upon several factors such as sample type, different stage of infection in patient, the skill of the collection, and the quality and consistency of the PCR-based diagnosis assays being used (38, 39). Therefore, the performance of PCR-based diagnosis may be false negative, resulting in clinical underdiagnosis. Our survey results showed that CT and epidemiological history are accepted by the majority of respondents. CT examination is not only convenient and quick, but also provides strong evidence, and is especially suitable for emergency and scheduled surgeries in inpatients and outpatients with suspected COVID-19. Chen et al. (40) considered hand sanitization as one of the most important measures to prevent epidemic-associated viral infections. Additionally, the (WHO) recommends hand decontamination by rubbing the hands with alcohol, for example, after glove removal (41). The above recommendations were generally endorsed by the respondents in the current study.

Aerosols are common in medical work environments, such as with nebulized inhalation and tracheal intubation, and samples of blood, urine, feces, etc. SARS-CoV-2 can be detected in aerosols for up to 3 h, on copper surfaces for up to 4 h, on cardboard for up to 24 h, and on plastics and stainless steel for up to 2 to 3 days (42). Guo et al. (43) analyzed workspaces in Wuhan Vulcan Hill Hospital (in the epidemic area) and found that SARS-CoV-2 was widely distributed in the air and on the surface of objects in the intensive care unit and general COVID-19 ward. Since the outpatient clinic room is an enclosed environment, staff and patients are susceptible to SARS-CoV-2 transmission by aerosols. In particular, physicians should raise the level of protection when contacting suspected or confirmed patients with COVID-19.

Xu et al. (44) found that some patients had a positive rectal swab even after a negative nasopharyngeal swab test. Therefore, when conducting digital rectal examinations in patients with suspected COVID-19 or recovering from COVID-19, it is recommended to do so gently and to wear double gloves, minimizing the occurrence of glove breakage. Some cases of post-operative death due to nosocomial infection of COVID-19 had been reported (45). Some studies had shown that pre-operative CT examination and nucleic acid testing are helpful to reduce the incidence of postoperative COVID-19 (12, 46). Electrosurgical and energetic devices should be used appropriately during outpatient procedures to promptly aspirate smoke and reduce aerosol injuries. During outpatient treatments or surgical procedures, physicians should use disposable instruments and other items as much as possible. For patients with suspected or confirmed COVID-19, devices used during the procedure, such as disposable instruments, sharp boxes, and catheters, should be clearly identified with a confirmed or suspected COVID-19 label and removed *via* the special infection channel (45, 46). Furthermore, Kuang et al. (46) suggested that clinical staff disinfect their soles before stepping out of the room of a patient with COVID-19.

The included flowcharts were standard procedures in our hospital, mainly based on the diagnosis and treatment protocol and technical guidelines of COVID-19 in China (9, 47). Although not officially standard, they were simple and easy to understand and could provide reference for outpatient protection work. If occupational exposure occurs during the above process, prompt symptomatic treatment is required. If body fluid exposure occurs, it is recommended that the operator should immediately remove the contaminant and repeatedly apply a large amount of saline rinse or 0.05% iodine for skin or mucous membrane rinsing and disinfection, respectively. In cases of blood exposure, the operator should gently squeeze the blood near the wound from the proximal end to the distal end, squeezing out as much blood as possible at the wound, followed by a rinse with flowing water, and disinfection with 75% alcohol or 0.5% iodine (12, 41). In the event of respiratory exposure, it is recommended to gently wipe the nasal cavity with a cotton ball containing 75% alcohol, followed by flushing with plenty of normal saline and medical isolation.

### Experience With Patients With a History of COVID-19 in Outpatient Clinics

Ma et al. (48) found significant changes in sex hormone levels in male patients with COVID-19 of reproductive age. Changes in sex hormones may affect future fertility (49). Our questionnaire recommended testicular and semen screening for fertility in men of reproductive age who have recovered from COVID-19. It may also provide new evidence on whether SARS-CoV-2 attacks the testes in the future. The majority of respondents in the current study indicated that outpatient urological control measures still need to be continued in the future in response to the domestic and international situation of the ongoing pandemic.

### Limitations

The survey and experiences were achieved from one specialty (urology) in one country (China), therefore the main results and conclusions may not apply to different settings or other regions. Considering that our knowledge of the disease was limited at the time of questionnaire formulation, this questionnaire study had some limitations. On some questions we limited the answer options, so that respondents might not be able to respond completely objectively. There were even more uncertainties when designing the questionnaire and responses, such as whether SARS-CoV-2 could be detected in semen and the possibility of transmission of SARS-CoV-2 through urine or semen. In addition, there were certain limitations in the sample size and survey distribution method of the questionnaire that may have affected the final dataset.

## Conclusions

Through the questionnaire survey, we learned about the current situation, experience and measures of epidemic prevention and control of COVID-19 in urology outpatient department in China. Although the scientific evidence of the survey was low, it could still provide us with a lot of helpful measure. The majority of physicians believed that SARS-CoV-2 could be detected in urine, and that protection against urine exposure was needed. These suggested flowcharts and recommendations to prevent new cases were accepted by most urologists in this survey. In terms of protective measures, it is still recommended to wear protection, especially when in contact with suspected samples during outpatient practice under the new normal of COVID-19.

## Data Availability Statement

The original contributions presented in the study are included in the article/Supplementary Material, further inquiries can be directed to the corresponding author/s.

## Ethics Statement

The studies involving human participants were reviewed and approved by The First Affiliated Hospital of Wenzhou Medical University and Taizhou Hospital of Zhejiang Province affiliated to Wenzhou Medical University, China. Written informed consent for participation was not required for this study in accordance with the national legislation and the institutional requirements.

## Author Contributions

H-HJ and MW had roles in conception and design. X-LZ, M-HJ, W-LL, J-HL, and Z-LS had roles in the questionnaire design, data collection, data analysis, data interpretation, and literature search. H-HJ and X-LZ had roles in writing of the manuscript. H-HJ, X-LZ, and MW contributed to the critical revision and data interpretation of the manuscript. MW had roles in supervision. All authors contributed to data acquisition, analysis, and interpretation, and reviewed and approved the final version.

## Conflict of Interest

The authors declare that the research was conducted in the absence of any commercial or financial relationships that could be construed as a potential conflict of interest.

## Publisher's Note

All claims expressed in this article are solely those of the authors and do not necessarily represent those of their affiliated organizations, or those of the publisher, the editors and the reviewers. Any product that may be evaluated in this article, or claim that may be made by its manufacturer, is not guaranteed or endorsed by the publisher.
